# Short-term results of a new self-locking cementless femoral stem: a prospective cohort study of the Lima Master^SL^

**DOI:** 10.1007/s12306-020-00651-1

**Published:** 2020-03-02

**Authors:** C. E. Dlaska, I. A. Jovanovic, A. L. Grant, G. Graw, M. P. Wilkinson, K. Doma, K. Hazratwala

**Affiliations:** 1 Orthopaedic Research Institute of Queensland, 7 Turner Street, Pimlico, Townsville, QLD 4812 Australia; 2 Department of Orthopaedics, Townsville Hospital, Townsville, Australia; 3 James Cook University, 1 James Cook Drive, Douglas, Townsville, QLD 4814 Australia

**Keywords:** Arthroplasty, Replacement, hip, Osteoarthritis, hip, Lima Master^SL^

## Abstract

**Background:**

Total hip arthroplasty is a successful treatment for hip osteoarthritis. Primary and secondary implant fixation is dependent on implant design and plays an important role in the longevity of an implant. In this study, we assessed the self-locking cementless Master^SL^ femoral stem.

**Materials and methods:**

In this single-centre prospective study, 50 consecutive hips with the indication for total hip arthroplasty, who met the inclusion criteria, received the Master^SL^ stem from LIMA Corporate. Patients had pre- and post-operative clinical and radiological assessment and completed patient-reported outcome measures [Oxford Hip Score (OHS), Harris Hip Score (HHS) and Forgotten Joint Score (FJS)] at the 6-week and 6-, 12- and 24-month mark. Post-operative X-rays were assessed for osteointegration (Engh Score), alignment and subsidence.

**Results:**

After 2 years, aseptic survival was 100%. One hip had to be explanted due to early deep infection and was excluded from the study. At 2 years, the patients reported a significant improved HHS and OHS of 95.3 ± 5.8 and 46.1 ± 3.6 (mean ± standard deviation), respectively, compared to preoperatively. The mean ± standard deviation for the FJS was 86.4 ± 18.7 with two-thirds of the patients reporting a score above 85. The mean Engh score is 15.1 ± 5.9 (mean ± standard deviation) with no patient scoring below 1 which suggests good osteointegration in all femoral stems.

**Conclusions:**

The Master^SL^ femoral stem performed well in this short-term follow-up study, with high patient satisfaction and good signs of osteointegration. Long-term follow-up will be necessary to evaluate longevity.

**Level of evidence:**

Level 3, Prospective cohort study.

**Trial registration:**

The study was registered on the 30.03.2016 with Australia New Zealand Clinical Trials Registry (ACTRN12617000550303).

## Introduction

Total hip arthroplasty (THA) has proven to be one of the most successful treatments in the field of orthopaedic surgery, even described as the “operation of the century” [1]. Over 1 million THAs are performed worldwide [2], and in Australia, it is one of the most common elective procedures undertaken with a 65% increase since 2003 [3]. THA is considered the gold-standard treatment for advanced osteoarthritis of the hip; it is highly successful in improving patients’ pain, achieving excellent functional results, and has a proven track record of long-term survivorship [4]. Despite these successes, some THAs need revision, and with the rising number of procedures performed, the number of revisions also rises. Although the reasons for revision are multifactorial, on-going improvements in implant design are necessary in order to reduce the burden of such procedures on both the patient and the healthcare system [5].

**Fig. 1:**
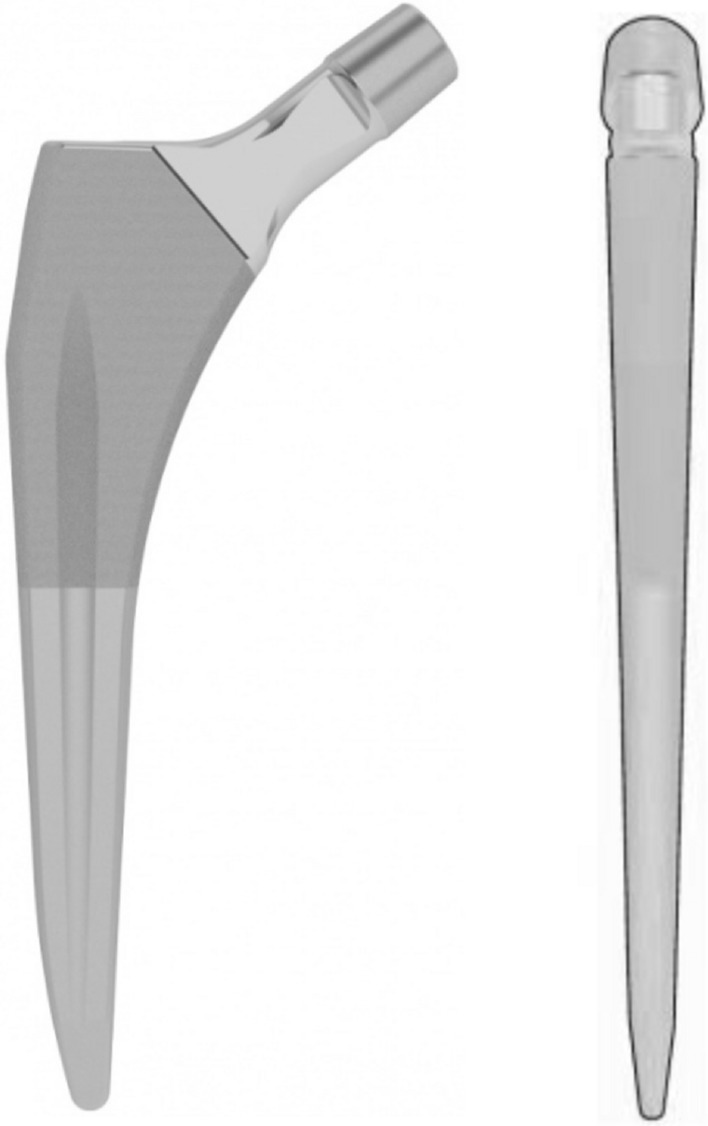
LIMA Master^SL^ femoral stem

Prosthetic hip implants have evolved over the past half a century, and contemporary technologies are driving innovation of improved prosthetic designs. When a new implant is introduced into the market, it is important to demonstrate that it is safe, reliable and easy for the surgeon to use with minimal to no learning curve. The new implant should produce at least equivalent but preferably superior results in terms of survivorship, alignment, complications and patient outcomes when compared to current available options. The Master^SL^ (self-locking) is a redesigned version of the existing SL femoral stem produced by LIMA Corporate, with changes to the anterior–posterior (AP) thickness and stem offset, which is proposed to produce a more anatomical reconstruction (Fig. 1). The collarless design of the implant allows full seating into the prepared canal (self-locking principle). The Master^SL^ system offers a greater variety of stem sizes, in both standard (neck-shaft angle: 131°) and lateralised (neck-shaft angle: 127.5°) offset. The sizing follows a linear increase in AP thickness, geometrical increment and offset. The proximal portion has a reduced lateral shoulder and the distal tip has been laterally rounded, to aid in minimally invasive surgical techniques and minimise cortical bone contact. The Master^SL^ femoral stem has a porous and hydroxyapatite-coated surface in the proximal half of the shaft for biological fixation and is designed for a broach-only technique.

The use of cementless femoral stems in THA was originally developed due to the high revision rates associated with poor cementing techniques [6]. Today’s cementing techniques and corresponding implants have largely improved; however, the difference in revision rates between cemented and cementless fixation is minor and the topic of many debates. Cementless fixation has its own benefits and disadvantages. A major concern with this type of fixation is bone resorption secondary to the stress-shielding effects of the cementless stem. This concern has led to the on-going developments to improved femoral stem designs such as the Master^SL^, which aims to reduce stress shielding on the cortical bone of the proximal femur. Furthermore, the redesigned Master^SL^ femoral stem is proclaimed to have increased stability within the femoral canal. These advancements have been proposed to improve and prolong the lifespan of the THA implants.

In this single-centre prospective study, two experienced surgeons (KH and MW) assessed the Master^SL^ femoral stem, which was new to the Australian market, and yet to have product registration at the commencement of the study period. The purpose of this study was to analyse the short-term survivorship, clinical and radiological outcomes and patient-reported outcomes in 50 consecutive patients who underwent a THA with the new Master^SL^ femoral stem, with a minimum 2-year follow-up.

## Materials and methods

For this single-centre prospective study, patients eligible for the study were provided with detailed information regarding their involvement, and written consent was obtained prior to the study commencing.

Between February 2014 and October 2015, every patient presenting to the outpatient clinic of surgeons KH or MW with osteoarthritis of the hip as an indication for a THA was considered eligible for the study. Exclusion criteria included women who were pregnant, children and/or young people (i.e. < 18 years), people with an intellectual or mental impairment, people highly dependent on medical care and people in existing, dependent or unequal relationships with any member of the research team. Sixty-seven consecutive potential participants were screened and 17 were excluded. From the eligible cohort, 50 consecutive patients were initially recruited; however, an early dropout due to insurance issues necessitated a further recruit. A total of 50 hips (out of 49 patients) were included in the final analysis.

The surgeries were performed by two experienced surgeons (KH and MW) at the same hospital. Neither of the two surgeons had used the Master^SL^ femoral stem before. Both surgeons were familiarised with the implant on a cadaver workshop provided by the company (LIMA). In addition, the surgeons operated together on three consecutive patients not included in the study, to gain more experience with the implant before the commencement of the study. Thereafter, each surgeon operated independently from the other 25 THA study cases. The posterior approach (Moore) [7] was used for all patients, and the surgical technique was as per the technique guide provided by LIMA Corporate, which utilises traditional instrumentation [8]. The preparation of the proximal femur after resection of the femoral head and neck included following steps, which were identical for both senior surgeons: with the box-osteotome, an entry to the femoral canal was created by removing some medial portions of the greater trochanter. The central canal reamer was used to determine the direction of the femur and with the curve rasp lateral cancellous bone was taken away. Sequential broaching was performed with a pneumatic broaching system until cortical fit medial and laterally was achieved.

The following pre- and post-operative patient-reported outcome measures (PROMs) were collected: The Harris Hip Score (HHS) [9] is a 100-point scale used to evaluate hip joint function, range of motion (ROM), pain and presence of deformities following THA; the Forgotten Joint Score (FJS) [10] asks the patient 12 questions regarding their awareness of their THA in everyday situations with a score ranging from 0 to 100 points; and the Oxford Hip Score (OHS) [11, 12] evaluates hip pain in everyday situations and the ability to do basic functional tasks with scores ranging from 0 to 48.

Follow-up was performed at the 6-week, 6-month, 1-year, and 2-year mark. It included clinical and radiological assessment and the completion of PROMs by the patient: HHS, OHS and FJS. The follow-up radiological images of the patients’ were assessed in regard to fixation (osteointegration) and stability using the Engh score [13] and analysed for subsidence and alignment. This was performed by another orthopaedic surgeon (CED), who was not part of the surgical team and was blinded to the clinical outcome of the patients. The Engh grading scale is the most prominent scale reported in the literature, a measurement scale that was first published in 1989 [13]. It contains two subscales, fixation and stability, which are summed for a total score. Based on the total score, the implant is classified into one of four categories: “unstable” (< − 10), “suboptimum but stable” (− 10 to < 0), “ingrowth suspected” (0 to + 10) and “bone ingrown” (> + 10) [14]. In regard to fixation, the X-ray is examined for lines, lucencies, and spot welds. The criteria for stability are the appearance of smooth interfaces, the presence of pedestal, calcar modelling, interface deterioration, migration and particle shedding.

The measure of central tendency and dispersion for all data was reported as mean ± standard deviation, or as frequencies for categorical data. Statistical analysis was performed with IBM SPSS ver.23.0 software. The alpha level was set at 0.05 for level of statistical significance. A paired t test was used to compare pre- (pre-op) and post-operative (2-years post-op) PROMs. To determine the magnitude of differences measures between time points, effect size (Cohen’s d) was also calculated with interpretation of < 0.5, 0.5–0.79 and > 0.8 considered as small, moderate and large, respectively.

## Patient demographics (Table 1)

A total of 50 hips (out of 49 patients) were included in the final analysis: 20 female and 29 male patients with a mean age at time of surgery of 64.7 ± 10.5 years (range 41–84 years), a body mass index (BMI) of 29.6 ± 5 (range 21–41) and a subjective assessment of the overall health of the patient with the score of the American Society of Anesthesiologists (ASA) of 2.1 ± 0.49 (range 1–3). All but one patient’s primary diagnosis for THA was idiopathic osteoarthritis; one patient (bilateral THA) had secondary arthritis due to Perthes disease.

**Table 1:** Patients demographics

Variable	Value
Age at time of surgery (years)*	64.7 ± 10.5 (41.3–84.1)
Gender	
Male	29
Female	20
Diagosis	
Primary OA	48
Secondary OA to Perthes	2
BMI (kg/m^2^)*	29.6 ± 5.0 (21–41)
ASA score (points)	2.1

*OA* osteoarthritis, *BMI* body mass index, *ASA* American society of anesthesiologist

*Values are expressed as mean ± standard deviation, with range in parentheses

## Implants

**Fig. 2:**
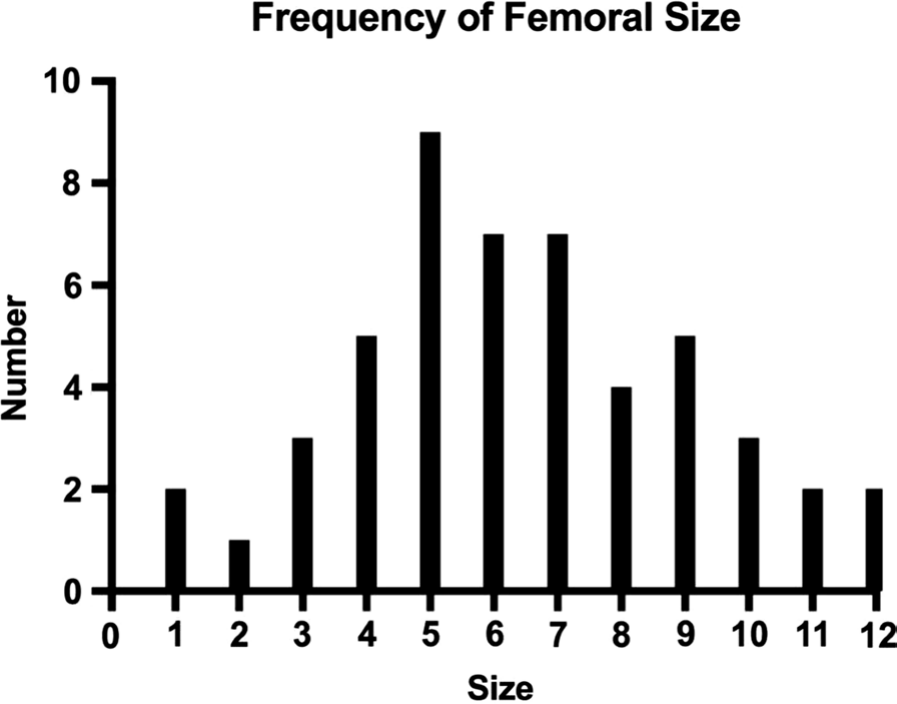
Frequency of femoral stem sizes used

All THAs were performed with the same Master^SL^ femoral stem (LIMA Corporate, Italy), in size and offset appropriate to the patient’s anatomy. The femoral stems utilised ranged from size 1 to size 12, with 42% (*n* = 21) receiving a lateralised stem. As such, almost the entire size range offered by the Master^SL^ system was utilised in the course of this study (Fig. 2). Femoral heads were either cobalt–chromium–molybdenum (CoCrMo) metal (36%, *n* = 18) or Biolox^®^delta ceramic (64%, *n* = 32). As the acetabular component, the Delta Primary System (LIMA Corporate, Italy) was utilised, with a trabecular titanium Delta-TT cup and either cross-linked polyethylene (60%, *n* = 30) or ceramic liners (40%, *n* = 20). For patients under 60 and with high functional demand, a ceramic head was combined with a ceramic or polyethylene liner.

## Results

### Functional and radiological outcomes (Fig. 3 and Table 2)

After the exclusion of one patient due to acute deep infection, two more patients were lost to follow-up. Therefore, a total of 46 patients (*n* = 47 hips) completed all assessments at the 2-year mark. Pre-operative HHS compared to at 2-year follow-up improved significantly from 50.3 ± 17.4 to 95.3 ± 5.8 (*p* < 0.001), and the mean OHS increased significantly from 22.4 ± 7.5 to 46.1 ± 3.6 (*p* < 0.001), respectively. The final HHS translates to 83% excellent, 13% good and about 4% fair results. Similarly, the OHS showed more than 91% excellent and about 9% good results. To detect any underlying bias of the investigator collecting the HHS, we compared the score improvements between the HHS and the OHS. No significant difference was seen (*p* = 0.215). The 2-year FJS reports a mean of 86.4 ± 18.7 with 36% (*n* = 17) of patients reporting the highest score of 100 points and two-thirds above 85 points.

**Fig. 3:**
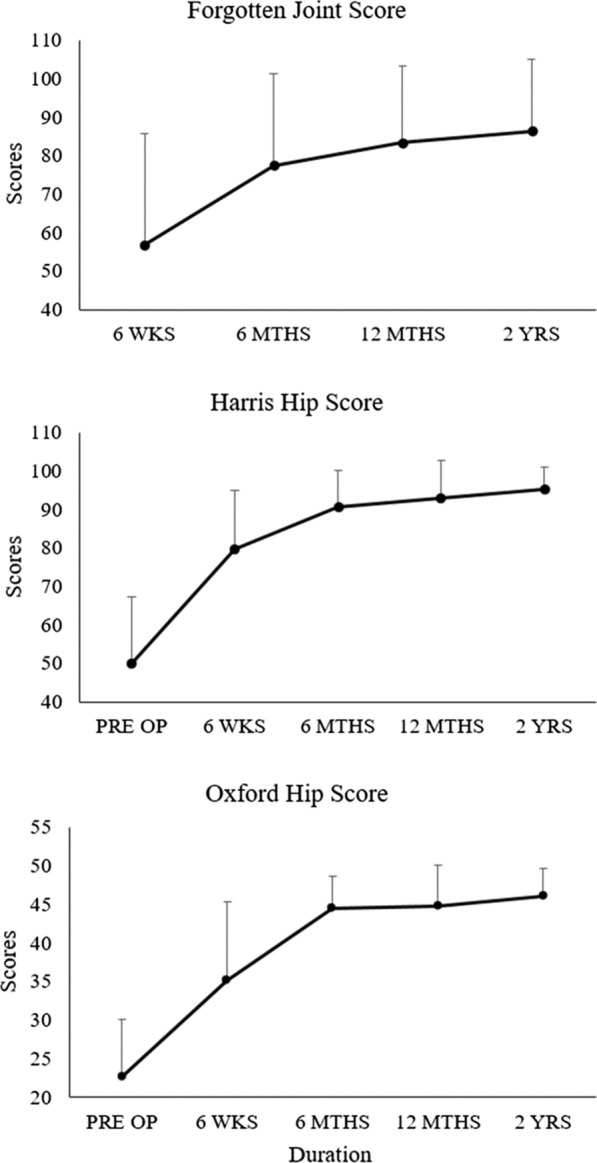
Mean PROMs at 6-week, 6-month, 12-month, and 2-year follow-up for FJS, HHS and OHS

**Table 2:** Functional outcome and radiological results

Variable	Value	
	Pre-OP	2-year follow-up
PROMs (*n* = 47)		
HHS*	50.3 ± 17.4 (2–87)	95.3 ± 5.8 (78–100)
HHS improvement*	45.1 ± 17.5 (7–86)	
OHS*	22.4 ± 7.5 (7–38)	46.1 ± 3.6 (30–48)
OHS improvement*	23.7 ± 7.7 (10–39)	
FJS*		86.4 ± 18.7 (20.8–100)
Radiological assessment (*n* = 47)		
Engh score*	15.1 ± 5.9 (1–27)	
Alignment*	0.43° varus (2.49° valgus—4.69° varus)	
Neutral (± 3°)	44	
Varus (> 3°)	3	
Valgus (> 3°)	0	
Subsidence		
Mean*	0.5 mm ± 0.8 (− 0.9–2.2)	
> 2 mm	1	

*PROM* patient-reported outcome measures, *HHS* harris hip score, *OHS* Oxford hip score, *FJS* forgotten joint score

*Values are expressed as mean ± standard deviation, with range in parentheses

The follow-up radiological assessment showed a mean Engh score of +15.1 ± 5.9, ranging from +1 to +27. 43 out of 47 hips had an Engh score higher than 5. The alignment of the prosthesis was neutral (± 3°) in 44, varus (range: 3–5.18°) in 3 and none were in valgus out of 47 hips. In regard to Engh’s assessment of subsidence, only one stem marginally breached the threshold of 2 mm when comparing the 6-week and 2-year follow-up X-rays. The mean subsidence measurement was negligible being 0.5 mm ± 0.8 mm (range: − 0.9–2.2 mm). One other femoral stem had a suspected change of alignment between the 6-week and 6-month X-rays from slight varus to neutral but with no signs of subsidence or any other signs of instability at the 2-year follow-up.

**Fig. 4:**
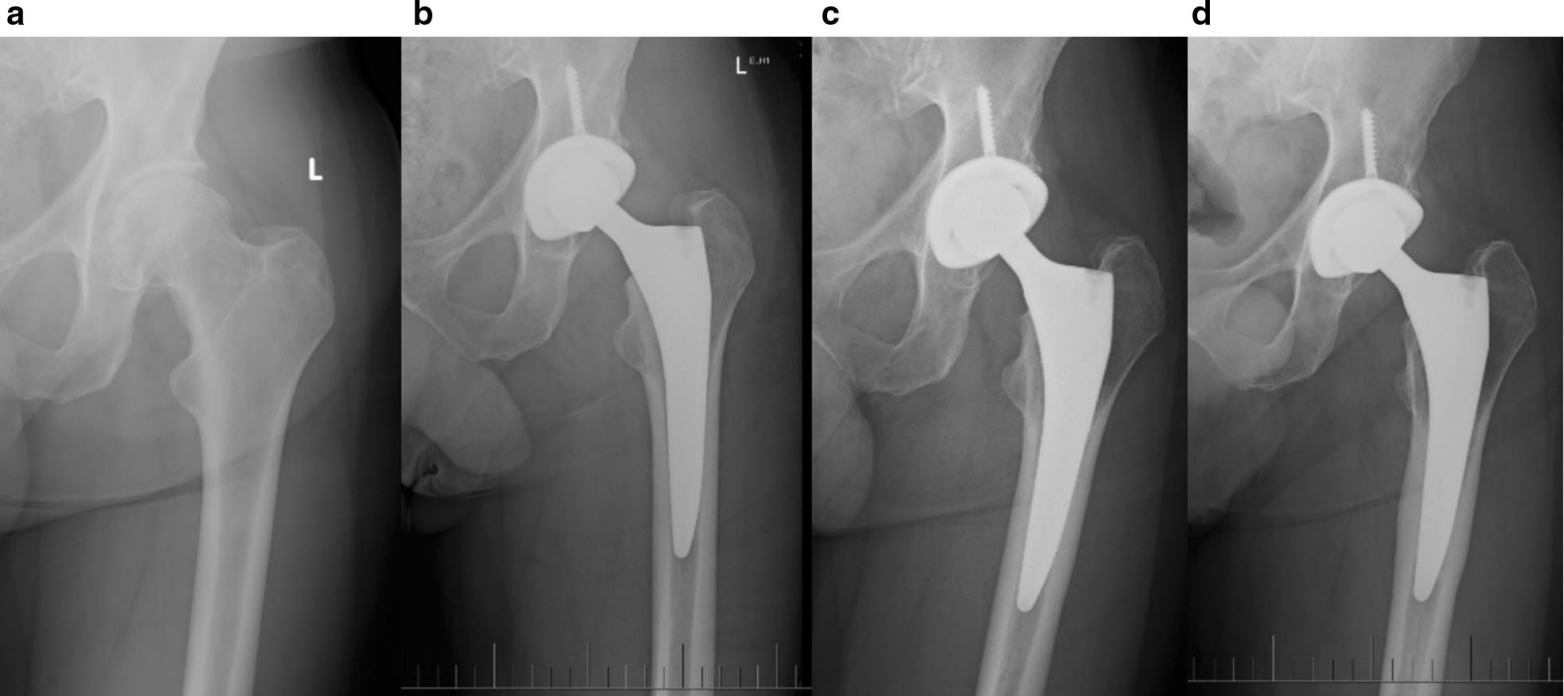
Diaphyseal thickening in 7 out of 47 cases. Example of a 69-year-old male patient with BMI of 25.7 kg/m^2^. Follow-up X-rays: **a** pre-OP, **b** 6 weeks, **c** 1 year, **d** 4 years

As part of the Engh score, we observed on the 2-year post-operative X-rays that the majority of the femoral stems had some lucency lines surrounding the smooth distal part of the stem. This is not unusual for stems with good metaphyseal fixation. Some other patients (*n* = 7) showed diaphyseal cortex thickening with more pronounced stress shielding proximally, suggestive of distal fixation of the stem in the diaphysis (Fig. 4). However, this subgroup of patients did not show statistically significant differences for FJS (*p* = 0.62), HHS (*p* = 0.85) and Oxford (*p* = 0.31) as the *p* values are well above 0.05. As for effect size, they came out as 0.26 (− 0.51–1.01) for FJS, 0.12 (− 0.64–0.88) for HHS and 0.54 (− 0.24–1.30) for Oxford. So, there were only small differences for FJS and HHS as they were below 0.5, although the difference in Oxford was moderate as it was above 0.5.

## Survivorship and complications

The aseptic survivorship of the Master^SL^ femoral stem after 2 years was 100%. Taking infections into consideration, we had one hip that failed and had to be revised. The complete implant of this joint had to be explanted due to deep infection (see below). No mechanical failure or aseptic loosening of any femoral stem was detected.

In this study, we had a total of five adverse events (Table 3), with one leading to the exclusion of the patient from the study. This patient had an acute deep infection 4 weeks post-implantation and required revision surgery with explantation of all implants and their components. Therefore, the patient was removed from the study. Another patient had a non-displaced longitudinal hairline fracture distal to the stem. The fracture was identified on the 6-week post-operative X-rays and was treated conservatively by partial weight bearing for 4 weeks. A computed tomography (CT) scan at 6 weeks post-operatively showed unchanged position of the stem with no evidence of subsidence and showed satisfactory callus formation. At 2-year follow-up, the fracture line was not visible on the X-ray and no subsidence was noted. The third patient developed a pulmonary embolism after surgery, which was confirmed on computed tomography of pulmonary arteries (CTPA). The patient was treated medically and recovered fully. The fourth patient had a fall on the ward on day 1 post-operatively sustaining a traumatic dislocation of his operated hip. An immediate relocation of the hip under anaesthesia was performed and the patient had no further events or complications. The last patient initially had an uneventful surgery but developed persisting pain and signs of loosening around the acetabular component 15 months post-implantation, resulting in revision of the acetabular cup. During the revision surgery, there was no sign of infection and only the acetabular cup was exchanged. The femoral stem was noted to be in the correct position without any signs of loosening and remained unchanged. Intraoperative samples were taken and processed for microbiology, and an infection was ruled out as cause of loosening of the acetabular component.

**Table 3:** Summary of complications

Complication	Number	Remark
Acute deep infection	1	Exclusion from the study
Intraoperative periprosthetic hairline fracture	1	Conservative treatment, no subsidence or loosening noticed
Pulmonary embolism	1	Conservative medical treatment
Early traumatic dislocation	1	On day 1, no further dislocations
Aseptic loosening of acetabular cup	1	Revision of cup, stem stable

## Discussion

Cementless fixation of the femoral stem in THA has shown great success especially in younger patients and is preferred by many experienced orthopaedic surgeons [15]. This type of fixation relies on good primary (mechanical) and secondary (biological) integration. Manufacturers address these important factors with specific THA implant design including materials, surface coatings and finishes. This study clinically investigated a redesigned femoral stem for THA, which was new to the Australian market and a refinement of a previously approved implant. THA is a successful and reliable treatment option for hip osteoarthritis with a vast variety of implants available on the market from multiple manufacturers. The Australian Joint Registry monitors the survivorship and revision rates for all THAs implanted in the country. Implants new to the market have to show their safety, longevity and survivorship over the years because surgeons will refrain from using implants that have higher than average revision rates.

This study shows the first early results of the new Master^SL^ femoral stem. The one major complication in this study, an acute deep infection, is unlikely to be related to the implant. The undisplaced hairline fracture identified in one patient is a complication seen occasionally with cementless femoral stems [15]. The surgeons in this study relate this complication to surgeon error rather than the implant itself.

The patients in this cohort have reported above average results in the HHS and OHS compared to the previously published literature [16–18]. In this study, excellent and good results in over 97% and 95% of patients were reported by the HHS and OHS, respectively, and fair was the worst score seen in this cohort.

The follow-up X-rays showed very good alignment of the femoral stem and a good fit in the metaphyseal and diaphyseal regions. Consistently, the surgeons were able to place the stem in the most desirable neutral position. The use of the Engh score to evaluate the fixation and stability of a cementless implant is of high value. Engh [13] was able to show that patients with scores above 5 were asymptomatic and had definite radiographic signs of bone ingrowth. Patients with positive scores between 0 and 5 had equally good clinical results but fewer radiographic signs of ingrowth [13]. In our study, all Engh scores were positive and more than 93% of patients had a score above 5.

Further evidence for good fixation is that no significant subsidence was measured on standard X-rays. Although X-ray measurements alone have limited accuracy, in conjunction with the positive Engh scores and good PROM results, it is suggested that all femoral stems had a high level of biological fixation. As part of the Engh score, we observed on the 2-year post-operative X-rays that the majority of the femoral stems had some lucency lines surrounding the smooth distal part of the stem. This is common for stems with good metaphyseal fixation. As mentioned in the results, seven patients showed diaphyseal cortex thickening which suggest distal fixation of the stem in the diaphysis (Fig. 4). The analysis of this subgroup and comparing it to all the other patients did not show any significant difference in their PROMs. Similar good results have also been seen with other tapered stems [19].

The short period of only 2-year follow-up is one limitation of this study. Generally, long-term survivorship of an implant and its performance requires 10–15 years of registry data. Therefore, further research with a longer period of investigation is required to establish implant survivorship and its potential functional benefits. Secondly, for assessment of fixation only serial X-rays were available which are less sensitive for assessment than CT or bone scan. However, considering the PROMs and clinical examination did not suggest any signs of loosening, and this study had only positive Engh scores, these more in-depth imaging modalities which are more costly and come with more radiation exposure would have not been justifiable.

The success of this new femoral stem in terms of survivorship and revision rates can only be properly evaluated with a larger cohort and longer-term data. The Australian joint registry will be a good tool to monitor the performance of this implant. In this small and limited study, the results are promising and show that the Master^SL^ femoral stem achieves similar short-term results to the best-performing implants available.

## Data Availability

The datasets used and/or analysed during the current study are available from the corresponding author on reasonable request.

## Abbreviations

OHS: Oxford hip score
HHS: Harris hip Score
FJS: Forgotten joint score
THA: Total hip arthroplasty
AP: Anterior–posterior
PROM: Patient-reported outcome measures
ROM: Range of motion
BMI: Body mass index
CoCrMo: Cobalt–chromium–molybdenum
TT: Trabecular titanium
CT: Computed tomography
CTPA: Computed tomography of pulmonary arteries
ASA: American society of anesthesiologist
