# Correction to: Short‑term results of a new self‑locking cementless femoral stem: a prospective cohort study of the Lima Master^SL^

**DOI:** 10.1007/s12306-020-00691-7

**Published:** 2020-12-31

**Authors:** C. E. Dlaska, I. A. Jovanovic, A. L. Grant, G. Graw, M. P. Wilkinson, K. Doma, K. Hazratwala

**Affiliations:** 1 Orthopaedic Research Institute of Queensland, 7 Turner Street, Pimlico, Townsville, QLD 4812 Australia; 2 Department of Orthopaedics, Townsville Hospital, Townsville, Australia; 3 James Cook University, 1 James Cook Drive, Douglas, Townsville, QLD 4814 Australia

## Correction to: MUSCULOSKELETAL SURGERY 10.1007/s12306-020-00651-1

The article Short‑term results of a new self‑locking cementless femoral stem: a prospective cohort study of the Lima Master^SL^, written by C. E. Dlaska, I. A. Jovanovic, A. L. Grant, G. Graw, M. P. Wilkinson, K. Doma, K. Hazratwala, was originally published electronically on the publisher’s internet portal on [02 March 2020] without open access. With the author(s)’ decision to opt for Open Choice the copyright of the article changed on 12 December 2020 to © The Author(s) 2020 and the article is forthwith distributed under a Creative Commons Attribution 4.0 International License, which permits use, sharing, adaptation, distribution and reproduction in any medium or format, as long as you give appropriate credit to the original author(s) and the source, provide a link to the Creative Commons licence, and indicate if changes were made. The images or other third party material in this article are included in the article’s Creative Commons licence, unless indicated otherwise in a credit line to the material. If material is not included in the article’s Creative Commons licence and your intended use is not permitted by statutory regulation or exceeds the permitted use, you will need to obtain permission directly from the copyright holder. To view a copy of this licence, visit http://creativecommons.org/licenses/by/4.0/.

The original article has been corrected.

